# iNaturalist accelerates biodiversity research

**DOI:** 10.1093/biosci/biaf104

**Published:** 2025-07-28

**Authors:** Brittany M Mason, Thomas Mesaglio, Jackson Barratt Heitmann, Mark Chandler, Shawan Chowdhury, Simon B Z Gorta, Florencia Grattarola, Quentin Groom, Colleen Hitchcock, Levi Hoskins, Samantha K Lowe, Marina Marquis, Nadja Pernat, Vaughn Shirey, Shukherdorj Baasanmunkh, Corey T Callaghan

**Affiliations:** Department of Wildlife Ecology and Conservation, University of Florida, stationed at the Fort Lauderdale Research and Education Center, Institute of Food and Agricultural Science Davie, Florida, United States; Evolution and Ecology Research Centre, School of Biological, Earth, and Environmental Sciences, University of New South Wales, Sydney; National Herbarium of New South Wales, Botanic Gardens of Sydney, Mount Annan, New South Wales, Australia; Centre for Ecosystem Science, School of Biological, Earth, and Environmental Sciences, University of New South Wales, Sydney, New South Wales, Australia; Department of Wildlife Ecology and Conservation, University of Florida, stationed at the Fort Lauderdale Research and Education Center, Institute of Food and Agricultural Science Davie, Florida, United States; Heifer International, Little Rock, Arkansas, United States; School of Biological Sciences, Monash University, Clayton, Victoria, Australia; Faculty of Environmental Sciences, Czech University of Life Sciences, Prague, Czech Republic; Centre for Ecosystem Science, School of Biological, Earth, and Environmental Sciences, University of New South Wales, Sydney, New South Wales, Australia; Faculty of Environmental Sciences, Czech University of Life Sciences, Prague, Czech Republic; Meise Botanic Garden, Meise, Belgium; Biology Department, Brandeis University, Waltham, Massachusetts, United States; Department of Wildlife Ecology and Conservation, University of Florida, stationed at the Fort Lauderdale Research and Education Center, Institute of Food and Agricultural Science Davie, Florida, United States; School of Natural Resources and Environment, University of Florida, Gainesville, Florida, United States; Department of Wildlife Ecology and Conservation, University of Florida, stationed at the Fort Lauderdale Research and Education Center, Institute of Food and Agricultural Science Davie, Florida, United States; School of Natural Resources and Environment, University of Florida, Gainesville, Florida, United States; Department of Wildlife Ecology and Conservation, University of Florida, stationed at the Fort Lauderdale Research and Education Center, Institute of Food and Agricultural Science Davie, Florida, United States; Institute of Landscape Ecology, Centre for Integrative Biodiversity Research and Applied Ecology, University of Münster, Münster, Germany; Marine and Environmental Biology Section, Department of Biological Sciences, University of California Los Angeles, Los Angeles, California; McGuire Center for Lepidoptera and Biodiversity, Florida Museum of Natural History, Department of Natural History, University of Florida, Gainesville, Florida, United States; Department of Biology and Chemistry, Changwon National University, Changwon, South Korea; Department of Wildlife Ecology and Conservation, University of Florida, stationed at the Fort Lauderdale Research and Education Center, Institute of Food and Agricultural Science Davie, Florida, United States

**Keywords:** citizen science, community science, participatory science, review, GBIF

## Abstract

Participatory citizen science is expanding, with iNaturalist emerging as one of the most widely used platforms globally. However, its application in research is often anecdotal. To evaluate the impact of how iNaturalist is contributing to biodiversity and conservation research, we conducted a systematic review of iNaturalist data use and compared our findings with Global Biodiversity Information Facility literature citing iNaturalist. We found that the use of iNaturalist data in peer-reviewed research has grown tenfold in the last 5 years, matching the growing increase in iNaturalist observations. Geographic and taxonomic representation in the literature generally aligns with data availability, with iNaturalist data derived from 128 countries and 638 taxonomic families being used in peer-reviewed literature. Currently, data from iNaturalist are primarily used for species distribution models and range dynamics. We highlight emerging trends in the use of iNaturalist data in the literature lending to its future potential across biodiversity sciences.

Participatory citizen science is increasing across diverse fields, including health and biomedical sciences (Wiggins and Wilbanks 2019), humanities (Tomić et al. 2021), planetary sciences (Odenwald 2018), environmental monitoring (Conrad and Hilchey 2011), and biodiversity science (Chandler et al. 2017). The number of projects and participants in biodiversity science is increasing exponentially (Pocock et al. 2017), in large part because of widespread internet availability and the ubiquity of smartphones capable of capturing high-resolution photographs: These technological advances empower nearly anyone to contribute valuable data (Land-Zandstra et al. 2016). Concurrently, advances in statistics, computer hardware, and software have expanded the analysis capabilities of scientists using these data. For instance, the toolkit for applying artificial intelligence to citizen science data is growing, allowing scientists to extract valuable information from large data sets, corresponding to an increasing rate of publications leveraging these data. At present, approximately seven articles are published per day, globally, using data from the Global Biodiversity Information Facility (GBIF) database (Ivanova and Shashkov 2021, GBIF 2025), from which more than 80% of the data since 2010 were derived from citizen science platforms (Callaghan et al. 2023).

In the face of unprecedented anthropogenically induced extinction rates, there is an urgent need for cost-effective methods to assess and document biodiversity (Cowie et al. 2022). Biodiversity data, such as those generated through citizen science initiatives, are playing an increasingly important role in providing the necessary data for biodiversity assessments (Chandler et al. 2017, Chowdhury et al. 2024, Gallagher et al. 2025). In addition, public involvement in biodiversity data collection increases public awareness and connection to conservation, likely resulting in greater support for conservation-related policies and environmental advocacy (Niemiller et al. 2021). Documenting and quantifying how biodiversity data are used not only helps evaluate the scientific impact but also enhances transparency and credibility of citizen science efforts. This can further engage the public, reinforcing the value of their contributions and fostering a deeper commitment to conservation initiatives through contributions to participatory citizen science initiatives.

## iNaturalist exemplifies a broad and varied biodiversity platform

We focus on one of the most popular global biodiversity-focused initiatives: iNaturalist (iNaturalist.org). iNaturalist is an independent, nonprofit organization that began in 2008 (Seltzer 2019) to “connect people to nature and advance science and conservation” (iNaturalist 2024). Participants upload photographs or audio recordings of any organism with the species name or lowest taxonomic level they can determine, as well as associated metadata such as location, time, and date (iNatHelp 2025). Once those assets are uploaded, other users can contribute taxonomic identifications to the observation at varying levels of taxonomic resolution. When more than two-thirds of the suggestions agree on the identified species and the observation passes the iNaturalist Data Quality Assessment (iNatHelp 2024), the observation is accepted as “research grade” and is added to GBIF following appropriate licensing requirements (Mesaglio and Callaghan 2021). The flexibility of the iNaturalist platform is such that it allows for the collection and curation of scientific information as both a byproduct of connecting people to nature and through more formal or structured opportunities facilitated by iNaturalist projects or observation annotations.

Among citizen science platforms that contribute biodiversity data, iNaturalist stands out for its scale, taxonomic breadth, and geographic coverage. As of September 2024, iNaturalist contained over 200 million unique observations from 3.3 million observers worldwide, making this platform one of the most successful on the basis of participation and data quantity (Mesaglio and Callaghan 2021). Within GBIF, iNaturalist stands out as one of the top contributors of global biodiversity data across all taxa, alongside Observation.org (Della Rocca et al. 2024). Although eBird contributes the largest volume of observations to GBIF, its data set is limited exclusively to birds. Compared with Observation.org, iNaturalist has better geographic coverage, considering that 97% of Observation.org observations are from Europe (GBIF 2025). Within GBIF, iNaturalist contributes the most observations for plants, mammals, reptiles, and amphibians, and ranks third for insect observations, following a United Kingdom–based macromoth distribution data set and Observation.org, although its numbers are close to those of Observation.org (GBIF 2025).

iNaturalist data are used in a range of topics in the biodiversity and conservation literature, especially to calculate species ranges and distributions (Fourcade 2016, Grattarola et al. 2023), describe species behavior and biology (Pernat et al. 2024), document and discover new species (Mesaglio et al. 2025), improve species classification (Van Horn et al. 2018), and quantify biodiversity shifts over time and in response to environmental change (Gorta et al. 2023, Grattarola et al. 2023). In addition, iNaturalist is used as an educational tool to increase biodiversity knowledge (Echeverria et al. 2021), data literacy, and biodiscovery (Hitchcock et al. 2021). iNaturalist plays a role in species conservation by contributing data that are used by the International Union for Conservation of Nature to monitor threatened species (Soroye et al. 2022, Gallagher et al. 2025). iNaturalist data are also used to document introductions of nonnative species (Hiller and Haelewaters 2019), inform invasive species management (Grattarola et al. 2024, Potgieter et al. 2024, Roger et al. 2024), and improve habitat suitability maps of nonnative species (Dimson et al. 2023). However, much of the current understanding of the breadth of iNaturalist's scientific contributions remains anecdotal, with limited systematic analysis of how extensively or consistently the data are applied across biodiversity and conservation research topics. A structured overview of the use of iNaturalist in the literature is necessary to fully assess its significance among topics and to determine geographic and taxonomic coverage of data and resulting impact on science, as well as guiding improvements in how the data are used in future research.

## A bibliometric analysis to comprehensively understand iNaturalist data use

Our objective was to understand the breadth of iNaturalist data use in the scientific literature. In conducting a comprehensive bibliometric analysis, we had three specific objectives: to quantify how the use of iNaturalist data is changing over time in the scientific literature and how that correlates with underlying patterns in iNaturalist observations, to assess the geographic and taxonomic coverage of research using iNaturalist data and test how that correlates with underlying patterns in iNaturalist observations, and to characterize the types of data, analyses, and topics researchers are engaging with when using iNaturalist data. To address these objectives, we synthesized peer-reviewed literature that used iNaturalist data or conducted a literature review of the platform. We then performed supplementary analyses by comparing our synthesis with GBIF-derived data.

### A workflow for aggregating iNaturalist articles

We searched for papers that used iNaturalist data in any capacity or provided an in-depth discussion on the iNaturalist platform and its applications (hereafter referred to as *iNaturalist literature*). Papers that only mentioned iNaturalist in passing, such as a brief mention in the paper's introduction or discussion, were excluded. We located papers with a combination of Google Scholar, Scopus, and the Web of Science. Google Scholar yielded the most results, because it provided full-text indexing, allowing us to locate papers where the use of iNaturalist was only mentioned in the methods (Ball-Damerow et al. 2019). Because of its unique name, we decided to run one search using the term “iNaturalist” across all search platforms. We recognize that there are different versions of the iNaturalist platform tailored to regional audiences, such as ArgentiNat (Argentina), NaturalistaUY (Uruguay), NaturalistaCR (Costa Rica), and NaturalistaCO (Colombia). These platforms are all part of the iNaturalist network, and approximately 75% of them include the term *iNaturalist* in their title, making them identifiable with our search term (iNaturalist 2025a). For the remaining platforms, which contribute research grade data to GBIF, we relied on information obtained from GBIF (see the “Supplemental analysis using GBIF” section below). Google Scholar searches all scholarly literature; however, for the present article, we were only interested in peer-reviewed articles, so we manually assessed the peer-reviewed status of each search result. We excluded non–peer reviewed literature from our review, such as conference proceedings, books, book chapters, technical reports, editorials, theses, dissertations, and data sets. Furthermore, we required articles to be freely available online or electronically available through our institutional access or by reasonable means. We included only articles written in English or Spanish, which captures a broad range of studies, because approximately 90% of natural science publications are in English, with a small additional proportion in Spanish (Hamel 2007).

**Figure 1.**
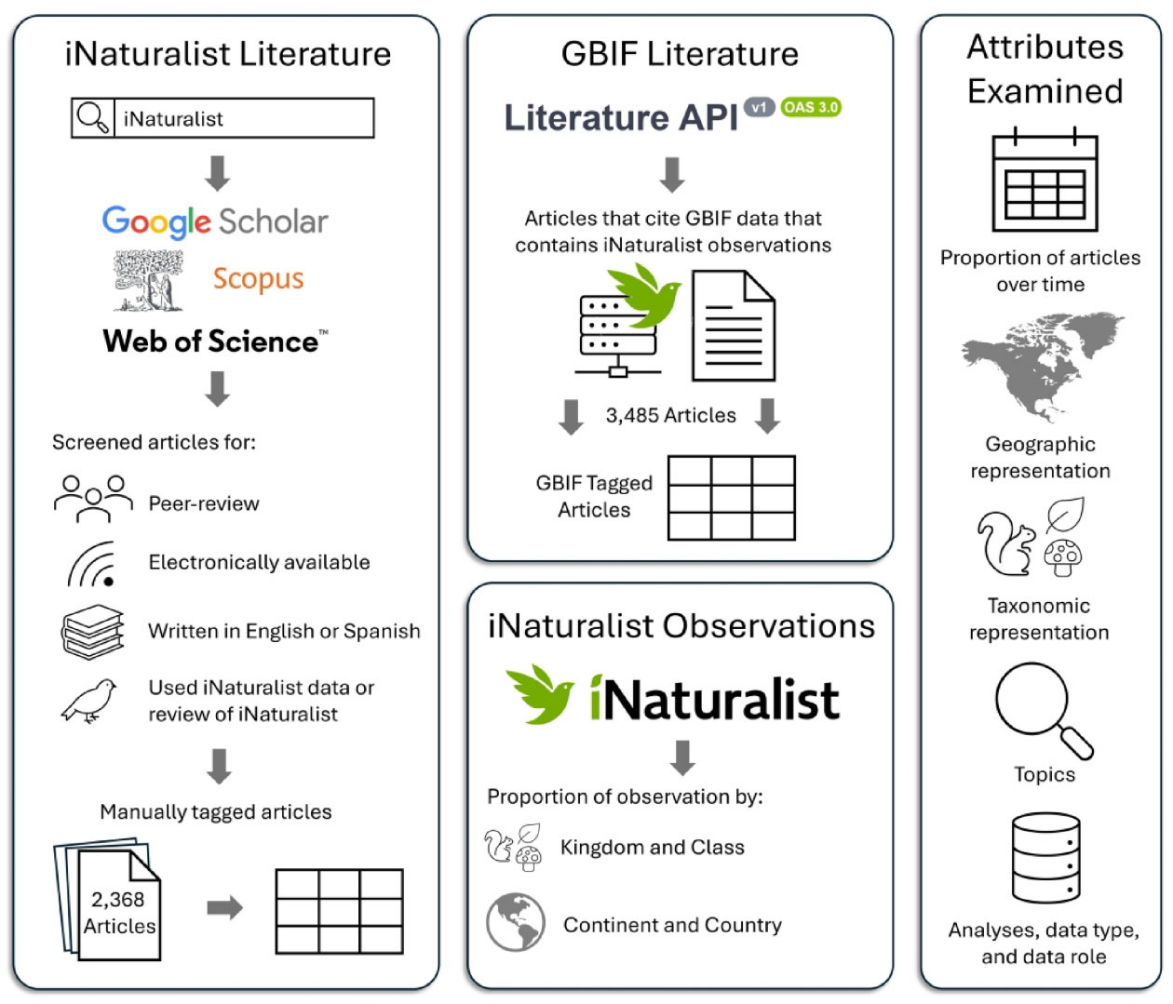
Methods for obtaining iNaturalist literature, Global Biodiversity Information Facility (GBIF) literature, and iNaturalist observations. The right panel displays the focus areas of this study.

We manually examined each article from the iNaturalist literature and tagged the geographic range, study taxa, species information, article topic, analyses conducted, iNaturalist data type, iNaturalist data role, and the use of project data (figure 1, see supplemental file S1 for the complete protocol). We categorized papers using iNaturalist data by topic (e.g., species distribution and range, biology and behavior, climate change and environmental impact; see supplemental file S1 for full list and definitions) to determine their popularity and identify underrepresented topics. These topics were chosen with the aim of capturing a wide range of iNaturalist applications while maintaining a manageable level of categorization. Although a finer-scale topic analysis could offer deeper insights into specific research areas, our focus was intentionally broad with the implication that future research can perform more granular classifications. The geographic range refers to the region covered by the iNaturalist data used in the paper, which may differ from the study's overall geographic scope. Similarly, we used each paper's methods section to determine the taxonomy of the data obtained from iNaturalist. If more than three taxa were represented within a specific taxonomic level, we consolidated them to the next finest taxonomic level that contained three or fewer taxa. For example, if a paper included butterflies and moths (order Lepidoptera), beetles (Coleoptera), flies (Diptera), and dragonflies (Odonata), we did not classify it at the order level because of the number of groups. Instead, we categorized it at a higher level: kingdom Animalia, phylum Arthropoda, and class Insecta. We also assessed whether each paper mentioned whether these taxa were species of conservation concern or classified as nonnative species but did not perform individual searches for each species outside of information presented in the paper. Finally, we assessed how often iNaturalist was the primary data source compared with being a supplemental data source that complements other open-source data or professionally collected data. Our protocol (supplemental file S1) provides detailed information on how our search was conducted among our authorship team.

### Supplemental analysis using GBIF

Because researchers can access iNaturalist data indirectly through GBIF without explicitly mentioning iNaturalist in their papers, its use may be underrepresented in the literature identified through our primary search. To address this, we supplemented our main analysis by examining literature indexed through GBIF to better understand the taxonomic and geographic distribution of iNaturalist data use. Specifically, we exported information on articles that used iNaturalist research grade data and cited them using a digital object identifier (DOI) via GBIF (hereafter referred to as *GBIF literature*) using the GBIF literature application programming interface (API). The GBIF literature data were downloaded in May 2024 and included literature from 2016 onward, when the DOI was first implemented. We did not manually examine all articles exported from GBIF, but rather relied on GBIF's designations of study region and taxa. To compare the overlap between the articles that mentioned iNaturalist and GBIF literature, we only used articles published after 2016 and prior to 2023, because we were unable to filter the GBIF literature beyond publication year. The GBIF literature provided information on study region and taxa, which we combined with the information we extracted from the iNaturalist review for the taxonomic and geographic analyses.

In addition, because GBIF aggregates biodiversity occurrence records from citizen science platforms and other public data sources, it serves as a valuable resource for tracking the growing contribution of iNaturalist to biodiversity research. To assess this, we examined iNaturalist's contribution to GBIF over time relative to other data set contributors by calculating the annual proportion of human observation records submitted by iNaturalist from 2010 to 2022. We focused on data through 2022 because data sets vary in how frequently they publish new records, and 2022 was the most recent year when the major data set contributors added their data. In addition, we calculated the proportion of observations that iNaturalist contributes to GBIF by taxonomic class for the 10 most frequently observed classes on iNaturalist on the basis of observation count.

### iNaturalist observations for comparison

To compare the taxonomic and geographic breath of articles to the available data on iNaturalist, we obtained a count of iNaturalist observations by kingdom and class taxonomy, country, and continent (iNaturalist 2025b).

### Data analysis and availability

All visualizations and empirical analyses were completed in R (R Core Team 2025) and relied heavily on the “tidyverse” ecosystem (Wickham et al. 2019). To find other possible trends in topics, we created a word cloud, using the R package wordcloud (Fellows 2018). We removed stop words, then created two sets of word frequency, one with unigrams and one with bigrams created using the R package tidytext (Silge and Robinson 2016). The two sets were combined, and we manually removed terms that overlapped with more informative multiword terms (e.g., *climate* was removed in favor of *climate change*). The resulting word cloud produced keywords that relate to the topics defined from our iNaturalist literature review.

To explore the relationship between the proportion of articles using iNaturalist data, from our iNaturalist literature review and GBIF, and the number of iNaturalist observations by country, we fitted a linear model using log-transformed proportions to achieve a normal distribution. From this model, we derived the residuals, representing the difference between the observed and expected proportion of articles, on the basis of the number of iNaturalist observations. We conducted this analysis for all countries with at least one article and separately for countries with at least five articles, because 53.3% of countries had fewer than five articles. Because the results were similar, we report findings for countries with at least five articles to minimize the influence of sparsely represented countries. A main objective of our work was to make the tagged data openly accessible, provided in supplemental table S1. To help allow for further visualization and exploration of how iNaturalist data are being used we also developed the Shiny app, which is viewable here: https://global-ecology-research-group.shinyapps.io/inaturalist-literature-review-shiny-app.

## iNaturalist usage is growing across the biodiversity sciences

**Figure 2.**
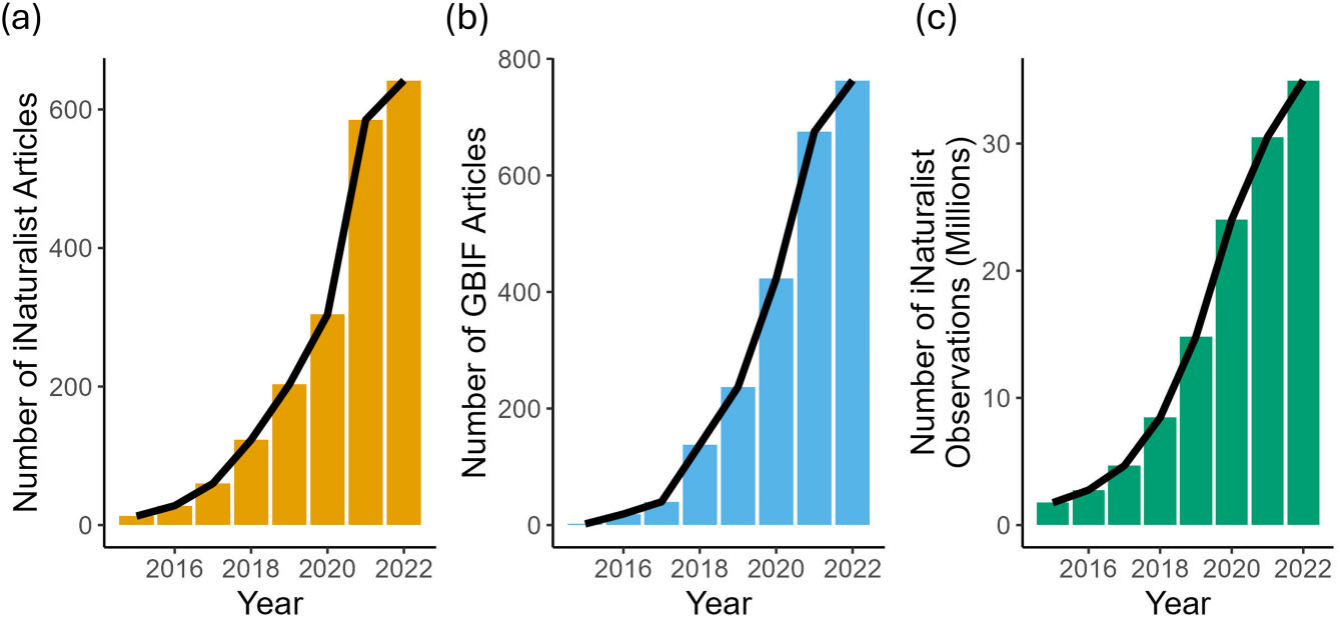
Count of scientific articles that use iNaturalist data over time from (a) iNaturalist literature and (b) Global Biodiversity Information Facility (GBIF) literature compared with the (c) count of iNaturalist observations on the platform over time. All data are shown for the years 2015–2022, the period during which all sources had at least one article and for which a full year of data was available.

After removing duplicate articles, we found that 98.3% of the articles were available from Google Scholar. Our initial literature search, conducted between 23 June and 12 July 2023, yielded 10,964 articles. Of these, 5914 results were peer-reviewed articles, and 2368 met our inclusion criteria. The GBIF literature API returned 3485 articles published up until 18 May 2024, the date the API was accessed. We found that only 10.3% of the articles from the iNaturalist literature search overlapped with the GBIF literature. Between 2015 and 2022, the proportion of iNaturalist and GBIF literature and iNaturalist observations grew exponentially (calculated as the annual number of articles or observations divided by the total number for each category; figure 2). In the last full year of data, 2022, 1410 articles used iNaturalist data.

When we compared iNaturalist's contribution to GBIF with those of other data sets, we found that the proportion of iNaturalist observations has increased over time (supplemental figure S1). Although the large contribution of eBird data reduces the proportion of iNaturalist contributions to 6.3% in 2022, when we exclude Aves from the calculation, we found that iNaturalist contributed more than a third of the observations in 2022 (37.2%). Among the 10 classes we examined, iNaturalist contributed the following proportions of observations in 2022: 55.1% for Amphibia, 63.3% for Arachnida, 54.9% for Gastropoda, 36.9% for Insecta, 33.1% for Liliopsida, 36.1% for Magnoliopsida, 24.9% for Mammalia, 51.1% for Polypodiopsida, and 74.0% for Reptilia.

### Geographic and taxonomic representation of scientific research using iNaturalist data

**Figure 3.**
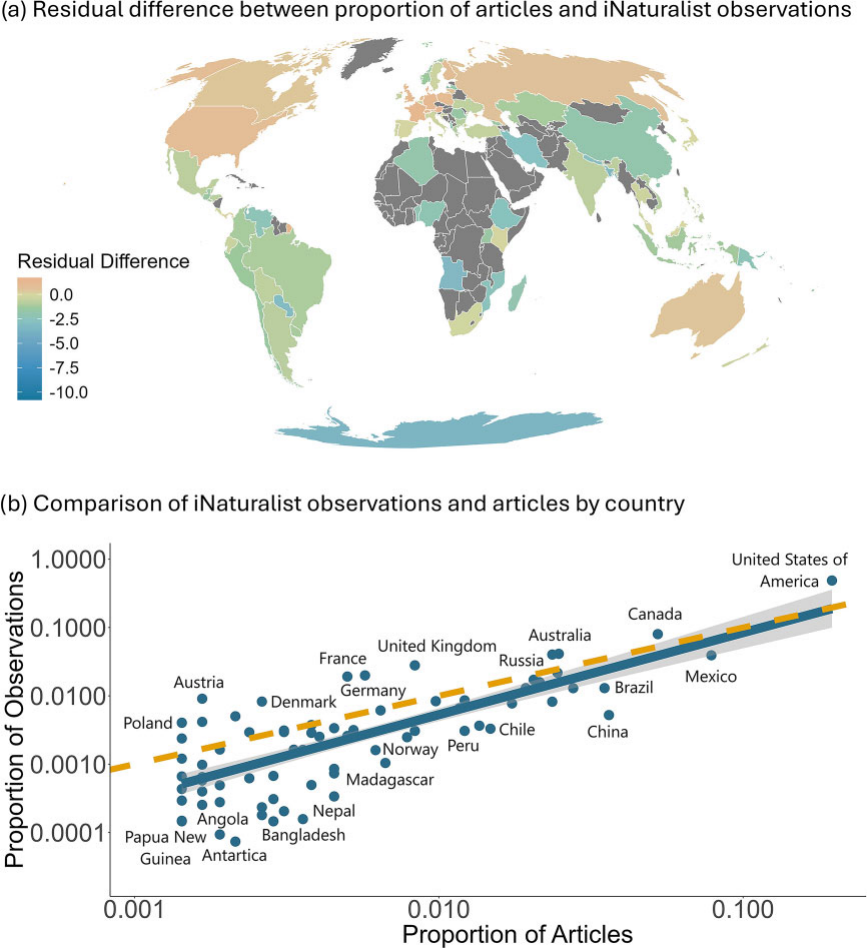
Comparison of the proportion of iNaturalist-related literature articles to iNaturalist observations, shown as (a) a map and (b) a scatterplot. The literature articles were sourced from iNaturalist literature and Global Biodiversity Information Facility (GBIF) literature. Countries with fewer than five literature articles were excluded because of lower confidence in those estimates. In the map, the regions with fewer articles than expected on the basis of the proportion of observations are denoted by positive residual difference values, whereas those with more articles than expected are denoted by negative residual difference values. The scatterplot is displayed on a log scale for both the ***x***- and ***y***-axes. The solid line represents the linear trend between the proportion of articles and the proportion of iNaturalist observations, with the shaded area indicating the standard error. The dashed line represents a 1:1 relationship, where the proportions are equal. The countries below the dashed line are overrepresented in the literature and above the dashed line are underrepresented in the literature. Selected points of interest are labeled. See supplemental figure S2 for the geographic distribution of iNaturalist literature, GBIF literature, and iNaturalist observations as a function of the country and a scatterplot of the proportion of articles from iNaturalist and GBIF literature as a function of the proportion of iNaturalist observations for each country. For detailed country-level data, see supplemental table S2.

In terms of geographic representation, we found that North America was the most represented region in terms of iNaturalist observations (55.9%) and study areas across the iNaturalist literature (39.1%) and GBIF literature (34.0%; figure 3). When comparing the proportions of literature study areas and iNaturalist data, we found that North America and Europe had fewer articles than expected given the number of iNaturalist observations, with residuals of 0.39 and 0.49, respectively. Contrarily, Africa, Asia, and South America had more articles than expected given the number of iNaturalist observations, with residuals of –1.32, –1.22, and –1.14, respectively. Oceania nearly had the expected number of articles given the number of iNaturalist observations, with a residual of 0.07. No studies from the iNaturalist literature and only six studies from our GBIF literature mentioned the use of iNaturalist data from Antarctica, likely because of the small proportion of iNaturalist observations from this region (0.0002%).

At the national scale, the United States had the highest proportion of study areas in the iNaturalist literature (28.4%) and GBIF literature (10.9%) and the highest proportion of iNaturalist observations (45.4%). We found countries such Bangladesh, Nepal, China, and Madagascar tended to have more articles than expected given the proportions of iNaturalist observations (figure 3, supplemental table S2). Contrarily, countries such as the United Kingdom, France, Australia, Canada, the United States, and Austria had fewer articles than expected given the proportion of iNaturalist observations (figure 3, table S2). We found that our linear model trend line between the proportion of articles and the proportion of observations started at a lower intercept and was steeper than the 1:1 ratio (figure 3b), indicating there is a stronger trend of countries that have a higher proportion of articles than expected given the number of iNaturalist observations.

The peer-reviewed literature used iNaturalist data from at least eight kingdoms, 57 classes, 638 families, and 1161 genera. Among all the studies, 51.5% were focused on three or fewer species. Of the GBIF literature containing taxonomic information (48.8% of the articles), iNaturalist data were used from eight kingdoms, 174 classes, and 7328 families. The larger number of classes and families from the GBIF literature is explained by the more comprehensive catalog of taxonomic groups sourced from iNaturalist in this data set, made possible by the citation of data exports.

**Figure 4.**
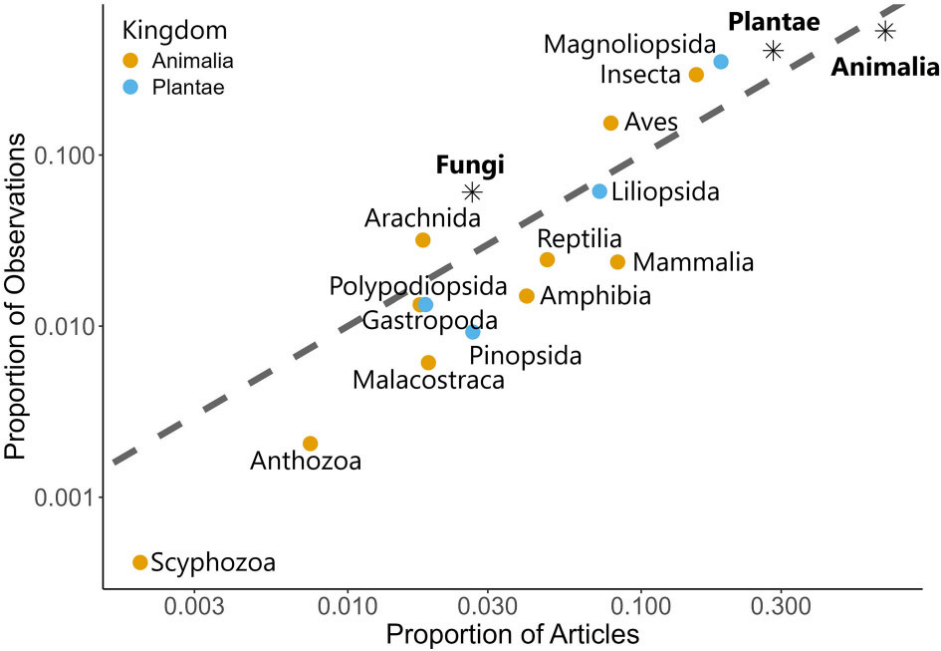
Taxonomic distribution of articles from iNaturalist literature review, Global Biodiversity Information Facility (GBIF) literature, and iNaturalist observations by kingdom (star symbol) and class (circle symbol) for Animalia and Plantae. Displayed are the groups that appeared in more than 5% of the articles in both literature data sets. The grey dashed line represents a 1:1 relationship, where taxonomic groups below the line are overrepresented in the literature and where those above the line are underrepresented in the literature. See supplemental figure S3 for bar plots of the proportion of each taxonomic group for each data source: iNaturalist literature, GBIF literature, and iNaturalist observations.

In both the iNaturalist and GBIF literature, Animalia was the most highly represented kingdom, consistent with being the most observed kingdom in the iNaturalist data (figure 4). This trend held true across all continents. However, compared with iNaturalist observations, iNaturalist articles had a higher proportion of Animalia represented in the literature (residual difference = –0.15) and a lower proportion of Plantae (residual difference = 0.13) and Fungi (residual difference = 0.03). In addition, we examined the top 10 Animalia classes and the top 4 Plantae classes, where each class represented over 5% of data in both literature data sets, compared with iNaturalist observations. Insecta had the highest proportion of study classes in the Animalia kingdom across all three sources but was underrepresented in the literature (residual difference = 0.14). In addition, there was an underrepresentation of Aves (residual difference = 0.07). Conversely, there was an overrepresentation of Reptilia (residual difference = –0.02), Mammalia (residual difference = –0.06), and Amphibia (residual difference = –0.02) in the literature. Compared with iNaturalist observations of Reptilia, Mammalia, and Amphibia, these taxa had higher representation in the literature. In the Plantae kingdom, Magnoliopsida (flowering plants) had the highest proportion of study classes across all three sources but was underrepresented in the literature (residual difference = 0.16). Liliopsida (monocotyledons; residual difference = –0.01) and Pinopsida (conifers; residual difference = –0.02), on the other hand, had higher representation in the literature.

### Characterization of the topics and data usage trends

**Figure 5.**
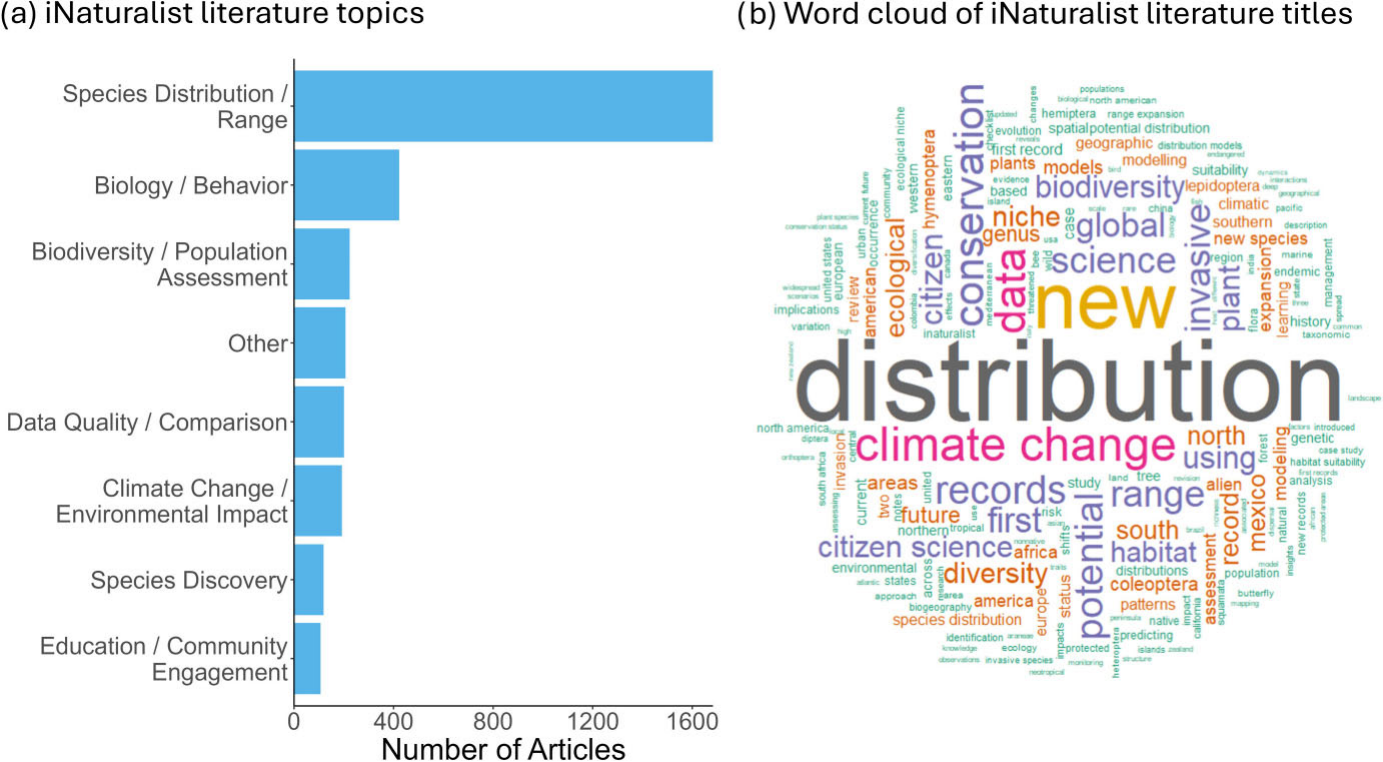
(a) The proportion of topics discussed in the literature from iNaturalist literature. The data are displayed from 2016 onward, when there were more than 15 articles represented in the iNaturalist literature review. In addition, the figure displays a (b) word cloud using unique titles from both the literature review and Global Biodiversity Information Facility literature. The larger words represent text that was more common in literature titles.

The most represented topic in the iNaturalist literature was species distribution and range (*n* = 1683), followed by biology and behavior (*n* = 423), biodiversity and population assessment (*n* = 223), other (*n* = 206), data quality and comparison (*n* = 200), climate change and environmental impact (*n* = 192), species discovery (*n* = 199), and education and community engagement (n = 107; figure 5a). The other topic primarily contained articles on image classification from machine learning using an iNaturalist image data set (48.6%), along with topics such as iNaturalist user behavior, discussions of iNaturalist projects, and discussions of the computer vision algorithm used by iNaturalist for identification suggestions. In general, there were no obvious temporal trends in topics or subject areas (supplemental figure S4). The word cloud of the paper titles in the iNaturalist and GBIF literature (figure 5b) supported these results. For instance, we found the term *distribution* to be common in the literature titles. Other terms of interest that were common are *new, climate change, conservation, biodiversity, range*, and *global*. In the iNaturalist literature, 393 articles (8.3% of all articles) reported using data on species of conservation concern and 536 articles (11.3%) on nonnative species. We found little positive co-occurrence associations among topics in the iNaturalist literature, because 70% of literature only fit within a single topic, supported by a co-occurrence analysis (see supplemental figure S5 for details).

**Figure 6.**
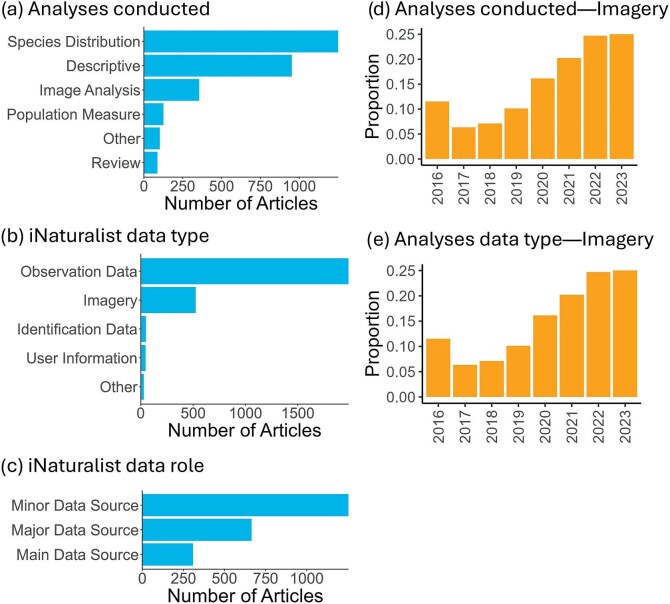
The count of articles grouped by (a) analyses conducted using iNaturalist data, (b) iNaturalist data type, and (c) iNaturalist data role in iNaturalist literature review papers. One article may fall into multiple categories of analysis type and data type. The proportion of articles using (d) imagery analysis and (e) imagery data type over time is displayed, as imagery showed the strongest trend among the categories examined (figure S6).

The most common analyses were species distribution (*n* = 1251) and descriptive analyses (*n* = 953; figure 6a). Few analysis types fit into the other category (*n* = 102), which consisted of regression analyses, network analyses, machine learning models, and fecundity analyses. We observed an increasing trend in image analysis and a decreasing trend in review papers (figure 6d, supplemental figure S6). We also categorized the types of iNaturalist data analyzed and found that observation data was the most frequently used category, accounting for 75.5% of the total (*n* = 1985; figure 6b). This is followed by imagery data (*n* = 524). The identification data (*n* = 48), user information (*n* = 45), and other data (*n* = 28)—which includes observation annotation data, project information, the iNaturalist taxonomic tree, and audio data—categories were used much less frequently. In the last 5 years, the proportion of observation data seems to have decreased, whereas the proportion of imagery data has increased (figure 6e, figure S6). We found that 1254 articles included iNaturalist as a minor data source, 665 as a major data source, and 308 as a main data source (figure 6c). There was no clear trend over time within these categories (figure S6). Only 241 articles (10.2% of all articles) mentioned they used iNaturalist data from an iNaturalist project.

In addition, we compared the distribution of topics, analyses conducted, and data role by taxonomic class. For all classes with more than 10 literature references from the iNaturalist literature review, the distribution of topics, analyses conducted, and data role within the classes were similar to the overall distribution of those categories with a few exceptions. Amphibia was more highly represented in articles that were focused on data quality and comparison (10.7% compared with the group average of 4.3%), and Magnoliopsida (11.5%), Liliopsida (8.5%), and Mammalia (10.6%) had higher than average (6.5%) representation in the climate change and environmental impact topic. Furthermore, articles that conducted an image analysis more often used images of Actinopterygii (ray-finned fishes; 19.1%) and Aves (18.6%) than the class average (10.6%).

## Future outlook and key takeaways

The use of iNaturalist data in the scientific literature is rapidly growing, with a more than tenfold increase in its presence in the literature in the last 5 years (2017 to 2022, when there was at least 50 articles), reflecting the platform's expanding data availability and increasing proportion of contributions to GBIF. For comparison, we found an increase of approximately 1.6 times the number of biodiversity related articles on Web of Science within this 5-year period, following the search methods of Stork and Astrin (2014). In 2022, we identified 1410 articles that used iNaturalist data, averaging nearly four articles published per day. This finding aligns with the growing trends observed in all of the GBIF literature citations over time (Heberling et al. 2021, Ivanova and Shashkov 2021). For instance, Heberling and colleagues (2021) found a twelvefold increase in the use of GBIF in the literature in 2007, and that approximately two articles using GBIF data were published each day in 2019. In addition, we found 638 taxonomic families in the iNaturalist literature and 7328 in GBIF literature, showing the breadth of taxonomic coverage.

### Strong correlation between number of publications and geographic and taxonomic data availability

Our analyses revealed a strong correlation between the geographic and taxonomic distribution of iNaturalist observations and their representation in the literature. This suggests that as iNaturalist data continue to grow across regions and taxonomic groups, the corresponding scientific literature is likely to expand as well. Encouraging people in underrepresented regions to use the platform is especially valuable as their contributions are particularly overrepresented in publications, helping to fill critical data gaps and enhance scientific output in those areas. In doing so, individuals may be empowered to contribute meaningfully to local conservation efforts.

We also identified some mismatches between the availability of iNaturalist data and its use in the literature. For instance, several countries such as Bangladesh, Nepal, China, and Madagascar that had an overrepresentation in the literature compared with the proportion of available iNaturalist observations. This may be due to these regions having high biodiversity (Farooq et al. 2020), which is more difficult to fully sample by professionals, leading to a higher use of citizens science data. Alternatively, the United Kingdom, France, Australia, Canada, the United States, and Austria had fewer articles than expected on the basis of their proportion of iNaturalist observations. Although this is somewhat surprising, given the potential bias toward English and Spanish language publications, we only considered the study region, not the geographic origin of the authors. It is therefore possible that researchers based in these countries focused their studies on other regions, relying more heavily on citizen science data collected abroad. In addition, these countries rank high on the Human Development Index (UNDP 2022), which may reflect greater funding for biodiversity research and increased availability of professionally collected data, potentially reducing reliance on citizen science sources. The residual values may also be a result of regional differences in methods detailing iNaturalist and GBIF citations (Luo et al. 2021).

Our taxonomic analysis similarly revealed mismatches between data availability and research attention. For example, our results highlight a greater proportional research focus on amphibians, mammals, and reptiles, whereas birds are comparatively underrepresented in the iNaturalist literature, likely because of the eBird citizen science platform, which contributes over 75 times the number of bird observations that iNaturalist contributes to GBIF (Sullivan et al. 2009, GBIF 2025). The difference between data availability and research interest on reptiles and amphibians has similarly been documented in a review of citizen science data usage (Feldman et al. 2021). Mammals may be overrepresented in research because of increased funding opportunities, which are often influenced by the charisma of species within this group (Gallo-Cajiao et al. 2018). Furthermore, mismatches between data availability and research attention may stem from differences in identification reliability. For example, some taxonomic groups, such as lichens, are more difficult to accurately identify from photographs (McMullin and Allen 2022), and scientists may regard citizen science data as less reliable for certain groups than others. We speculate that these differences may lead to the over or under representation of the use of iNaturalist data in publications, both taxonomically and geographically, which is an important area of future research to better understand the processes driving iNaturalist data use in publications.

### Species distribution, biological traits, and emerging research trends

The most common use of iNaturalist data is to describe and predict species distributions and ranges. This corroborates the documented increasing rate of species distribution models that use citizen science data, which is increasing at double the rate of overall species distribution model papers (Feldman et al. 2021). This trend may be attributed to recent methodological developments in statistics such as integrated species distribution models, which can be used to integrate presence-only iNaturalist observations with other data types, such as presence or absence data or data from standardized collection methods, to robustly calculate species distribution (Isaac et al. 2020, Grattarola et al. 2023). Within the species distribution or range topic, we observed several themes, including studies addressing species range expansion, records of nonnative species in new regions, and regional species checklists. Future research could further examine articles in this area to fully understand the extent of these applications.

iNaturalist data have been applied in diverse research areas beyond species occurrence, offering valuable insights into biological traits, population assessments, climate impacts, and species discoveries. In this study, we found that 12.3% of the included papers documented biological traits of species such as diet (Nupen et al. 2023), pigment variation (Tseng et al. 2022), and behavior, including plant–pollinator interactions (Fontúrbel et al. 2023) and breeding behavior (Díaz et al. 2023). In addition, 7.5% of the articles conducted a population assessment of a species, including studying the abundance of flowering plants and specialist bees (Smith et al. 2023). Another 5.5% of the articles examined climate change, such as its impact on habitat suitability of sensitive bird species (Scridel et al. 2021) and environmental impact, such as urban heat island effect on wildlife activity (Herrera and Cove 2020). Furthermore, 3.7% of the articles used iNaturalist data to document new species, such as the discovery of a new species of gall wasp from an image that was first shared on iNaturalist (Zhang et al. 2022).

Image analyses using iNaturalist data increased over time and could surpass the other data analysis types soon. This aligns with the iNaturalist data type that was used in the papers, where we found that a decreasing proportion of the papers used observation data and an increasing proportion of the papers used imagery data. This may be explained by a recent increase in literature surrounding machine learning analyses on image data sets, opening more possibilities for analysis on iNaturalist imagery data sets and secondary data (Pernat et al. 2024). The rise in the use of imagery data suggests that the quality and type of imagery uploaded to iNaturalist should be considered not only for species identification but also for extraction of information on attributes such as habitat, species coloration, and behavior. Metadata on images, such as annotations made on the iNaturalist platform, may further encourage the use of iNaturalist imagery in the literature. Therefore, citizen scientists should upload high-quality images and consider the metadata that could be extracted from images (i.e., pollinator–plant interactions, habitat information, interaction behavior) when collecting observations. In addition, the iNaturalist community should provide annotation information to observations when they are relevant. Scientists should also communicate their secondary data needs to the iNaturalist community to ensure alignment. In addition, we observed a decrease in the proportion of review papers, which, in the early years of iNaturalist, were focused on the novelty of the platform compared with other similar platforms. However, as iNaturalist became more popular and the data set grew, this opened more analytical possibilities to empirically advance many biodiversity questions. For instance, we found that 44.1% of the articles relied on iNaturalist to fully or more robustly answer research question, highlighting its importance in biodiversity research.

The distribution of topics, analyses conducted, and iNaturalist data role by class often aligned with the overall distribution among all classes, with a few exceptions. The slightly higher proportion of articles focusing on Amphibia and data quality or comparison may be attributed to the cryptic nature of some amphibian species and the significant declines in amphibian populations, with 32.5% of amphibian species classified as threatened (Köhler et al. 2005, Lee et al. 2021), resulting in researchers taking a stronger interest in the data availability and quality in relation to amphibians. For instance, amphibians tend to take cover under leaf litter or partially or fully submerge underwater, making a full checklist of amphibians in a region from citizen science data challenging, although necessary given the decline in amphibian populations. This is evidenced by articles comparing iNaturalist species checklists with natural history museum checklists (Duran 2021) or AmphibiaWeb (Forti and Szabo 2023). In addition, amphibian contributions to iNaturalist may include audio recordings of frog calls, which require their own data quality assessment. The higher proportion of studies on Magnoliopsida, Liliopsida, and Mammalia in climate change and environmental impact research follows previously documented trends within these taxa. For instance, Pacifici and colleagues (2015) found that mammals and plants were the second and third most analyzed taxon in climate change research, after birds. The above-average use of image analysis in Actinopterygii and Aves studies is likely driven by research on coloration (Aguillon and Shultz 2023, Nyegaard et al. 2023), fish identification (Valente et al. 2021), and bird diet (Sandvig and Cerpa 2022) and breeding behaviors (Tubelis and Dornas 2021), all of which rely on imagery.

### Comparison of iNaturalist and GBIF approach

Our comprehensive review uncovered an additional 1914 articles not captured by GBIF, underscoring the value of manual tagging for a thorough understanding of iNaturalist's scientific reach. This is probably because not all researchers properly cite the GBIF DOI (Heberling et al. 2021), which should be encouraged to make reviews of iNaturalist data usage more efficient and accessible. In addition, researchers do not always use GBIF to access iNaturalist data, but access them directly through iNaturalist, including the iNaturalist API, or make direct or indirect references to single iNaturalist observations. This finding highlights the importance of incorporating manual tagging and comprehensive bibliometric approaches to fully capture the breadth of scientific contributions from citizen science platforms, ensuring that their true impact on biodiversity research is accurately reflected and not underestimated by automated citation tools alone. However, as the body of iNaturalist literature continues to grow, having expanded significantly from the time of tagging to the time of this publication, manual tagging will become less feasible. Therefore, we recommend that researchers explore the use of machine learning to tag articles. This approach would enable more efficient analysis of the expanding iNaturalist literature (Atkinson 2024) and provide adaptability to evolving research directions. To help facilitate such approaches in the future, the tagged data have been made available (table S1; Shiny app: https://global-ecology-research-group.shinyapps.io/inaturalist-literature-review-shiny-app/).

### Future of iNaturalist in biodiversity research

iNaturalist data are primarily used for species distribution and range mapping, which highlights one of the strengths of this data source. For example, iNaturalist data have been demonstrated as a suitable means to map global trends in plant functional traits, and they are expected to play an increasingly significant role in this realm (Wolf et al. 2022). The increasing use of iNaturalist imagery data opens the potential to study species biology on a larger scale. For example, iNaturalist imagery and metadata can generate high-quality spatial phenotypic data, expanding the geographic and temporal scope of studies compared with what was known previously (Drury et al. 2019). iNaturalist can also provide critical data on poorly monitored species, thanks to the contributions of both contributors and experts. This has led to the documentation of many first known photographs of thousands of species, primarily in regions that are underrepresented in expert monitoring (Mesaglio et al. 2021). In conjunction with the innovative uses of iNaturalist data, researchers have come up with strategies to increase the applications of these data. For example, researchers have developed a model that considers the observation of the target species along with the number of nontarget species the observer recorded as a measure of pseudoabsence with additional predictor variables. This method results in more accurate species distribution models compared with the previous method of simulating random pseudoabsences (Milanesi et al. 2020).

Although iNaturalist hosts an extensive biodiversity data set, it also has limitations. Notably, iNaturalist observations exhibit spatial, temporal, and taxonomic biases (Hochmair et al. 2020, Di Cecco et al. 2021, Rosa et al. 2022), which we found in this article. Some researchers have addressed this issue by actively recruiting local communities to contribute to iNaturalist projects, which are designed to encourage observations of particular taxa and regions (Garrido-Priego et al. 2023, Terenzini et al. 2023). However, such targeted recruitment remains relatively uncommon, with only 10.2% of the articles explicitly referencing project-specific data or mentioning specific participant recruitment strategies. One avenue to encourage additional participation is through bioblitz activities such as the City Nature Challenge, which is a friendly worldwide competition where cities host events to document biodiversity on iNaturalist. This event has successfully increased observation numbers on iNaturalist and can be leveraged by countries with a lower proportion of iNaturalist observations (Palma et al. 2024). In addition, there are geographic and taxonomic biases in the number of observations that are likely to reach research grade status (Campbell et al. 2023). A major potential to help alleviate this problem is the increased uptake and encouragement of identifications by experts on the iNaturalist platform, which can help open more research possibilities and continue to fill geographic and taxonomic gaps (Callaghan et al. 2022).

Beyond the biases in the iNaturalist data set, we observed potential biases in the scientific literature. For instance, we observed a bias in the reliance on citizen science by country, with countries such as China, Madagascar, Nepal, and Bangladesh relying more heavily on citizen science than countries such as France, Germany, the United Kingdom, and Australia. Moreover, there appears to be a bias by researchers toward classes such as Mammalia, Reptilia, and Amphibia. Nevertheless, the growing documentation of these biases (e.g., Di Cecco et al. 2021, Campbell et al. 2023) is paving the way for scientists to address them through analytical techniques, targeted recruitment of iNaturalist contributors, or supplementary field sampling in areas where iNaturalist data are insufficient. Although having more data available on a variety of taxon on iNaturalist invites researchers from various disciplines to use the data, there likely will continue to be biases toward certain study taxa depending on scientific interest and government needs. Given that long-term participants in citizen science tend to develop more collaborative motivations (Rotman et al. 2014), researchers could leverage this by clearly communicating their data needs to their participants. This may help increase the proportion of iNaturalist observations that are most relevant to their studies.

## Conclusions

iNaturalist data have grown rapidly and continues to attract increasing attention year after year. Although iNaturalist's mission is to “connect people to nature and advance biodiversity science and conservation” (iNaturalist 2024), the extent to which the resulting scientific literature uses iNaturalist data was previously unclear. We showed that iNaturalist generates data that support research spanning multiple disciplines (e.g., education, machine learning, conservation, taxonomy, and ecology), geographic regions, and taxonomic groups. Our iNaturalist literature review revealed representation across 128 countries and 638 taxonomic families, highlighting the significant role iNaturalist is playing in biodiversity research. iNaturalist is helping to democratize data collection, opening new opportunities for studies that were previously limited by geographic or taxonomic data gaps. We encourage researchers and stakeholders to continue to promote public engagement in underrepresented regions and taxa. Our findings offer a clear and comprehensive view of how iNaturalist data are already being used across the biodiversity sciences, providing a valuable tool for stakeholders to communicate the scientific impact of the platform. This visibility can help reinforce a positive feedback loop, where participants and researchers alike are further motivated to contribute, use, and enhance the quality of iNaturalist data. By actively encouraging users to contribute more observations and species identifications, the scope and utility of iNaturalist data will continue to grow, increasing the potential for biodiversity research. In addition, innovations in confidence scoring of iNaturalist records could improve data quality and reduce the time it takes for observations to become verified (Ackland et al. 2024). iNaturalist is often used alongside other data sources, so standardized data collection remains essential and works synergistically with iNaturalist data for biodiversity studies. Our work provides a snapshot of current iNaturalist data use, highlighting its taxonomic and geographic breadth and its growing impact on biodiversity research.

## Supplementary Material

biaf104_Supplemental_Files

## Author Biography

Brittany M. Mason (bmason1@ufl.edu), Jackson Barratt Heitmann, Levi Hoskins, Samantha K. Lowe, Marina Marquis, and Corey T. Callaghan (c.callaghan@ufl.edu) are affiliated with the Department of Wildlife Ecology and Conservation at the University of Florida, stationed at the Fort Lauderdale Research and Education Center, Institute of Food and Agricultural Science, in Davie, Florida, in the United States. Thomas Mesaglio is affiliated with the Evolution and Ecology Research Centre, in the School of Biological, Earth, and Environmental Sciences at the University of New South Wales, in Sydney, and with the National Herbarium of New South Wales, Botanic Gardens of Sydney, in Mount Annan, New South Wales, in Australia. Mark Chandler is affiliated with Heifer International, in Little Rock, Arkansas, in the United States. Shawan Chowdhury is affiliated with the School of Biological Sciences at Monash University, in Clayton, Victoria, Australia. Thomas Mesaglio and Simon B. Z. Gorta are also affiliated with the Centre for Ecosystem Science in the School of Biological, Earth, and Environmental Sciences at the University of New South Wales, in Sydney, New South Wales, Australia. Shawan Chowdhury and Florencia Grattarola are affiliated with the Faculty of Environmental Sciences at the Czech University of Life Sciences, in Prague, in the Czech Republic. Quentin Groom is affiliated with the Meise Botanic Garden, in Meise, Belgium. Colleen Hitchcock is affiliated with the Biology Department at Brandeis University, in Waltham, Massachusetts, in the United States. Levi Hoskins and Samantha K. Lowe are affiliated with the School of Natural Resources and Environment at the University of Florida, in Gainesville, Florida, in the United States. Nadja Pernat is affiliated with the Institute of Landscape Ecology and with the Centre for Integrative Biodiversity Research and Applied Ecology, at the University of Münster, in Münster, Germany. Vaughn Shirey is affiliated with the Marine and Environmental Biology Section of the Department of Biological Sciences at the University of California Los Angeles, in Los Angeles, California, and with the McGuire Center for Lepidoptera and Biodiversity, at the Florida Museum of Natural History, under the Department of Natural History at the University of Florida, in Gainesville, Florida, in the United States. Shukherdorj Baasanmunkh is affiliated with the Department of Biology and Chemistry at Changwon National University, in Changwon, South Korea.
