# Supplementary material for: iNaturalist accelerates biodiversity research
Source: Bioscience. 2025 Jul 28;75(11):953–65. doi: 10.1093/biosci/biaf104 (PMC12650526; doi:10.1093/biosci/biaf104)
Supplement: biaf104_Supplemental_Files [file biaf104_supplemental_files.zip › Supplementary_File_1_Mason_et_al.docx]

**Supplementary File 1**

In this document, we detail the database fields, and the protocol for tagging each field. To ensure clarity of the protocol, all co-authors reviewed the protocol and tagged ten articles that had already been tagged by the lead author. The lead author then compared these tags with their own and met with each co-author to resolve any misunderstandings in the protocol. After this meeting, co-authors proceeded to tag articles independently but were instructed to flag any articles they were uncertain about. The lead author then manually reviewed these flagged articles.

**Database fields**

**Key:** Unique key created by Zotero

**Language:** Language of the article – English or Spanish. All other languages were excluded from this study.

**Search engine:** The search engine where the article was initially found.

**Publication year:** The year the article was published.

**Author:** Author(s) of the article.

**Title:** Title of the journal article.

**Journal:** Journal where the article was published.

**Pages:** Pages of the article in journal.

**Issue:** Journal issue.

**Volume:** Journal volume.

**Notes from screening:** Notes made by reviewer during the screening process.

**Screened by:** Person assigned to determine article relevancy.

**Study area region:** If applicable, reported region of study area. If multiple regions are described, separate them with a comma and a space unless more than three regions are described. In this case, just define to country level.

**Study area country:** Reported country of study area. If multiple countries are described, separate them with a comma and a space unless more than three countries are described. In this case, just define to continent level. If study area country is not explicitly stated but can easily be inferred by study species range or figures, you may report this country. However, if it is too time consuming to determine the country the study took place, leave this field blank.

**Study area continent:** Reported continent of study area. If multiple continents are described, separate them with a comma and a space. If the study uses all iNaturalist data or is global in scope, record “global.” If study location is not specified, leave this field blank. Please reference the continent map from the World Population Review to help with continent designation as not all sources define the same countries to the same continents.

**Studied taxa - kingdom:** If applicable, the kingdom of the taxa studied from iNaturalist taxonomy. If multiple, list all kingdoms, separated by a comma and space. If study uses all iNaturalist data, leave this field blank.

**Studied taxa - phylum:** If applicable, the phylum of the taxa studied from iNaturalist taxonomy. If multiple, list all phylums, separated by a comma and space. Unless phylum list is greater than three, in this case just define taxa to kingdom.

**Studied taxa - class:** If applicable, the class of the taxa studied from iNaturalist taxonomy. If multiple, list all classes, separated by a comma and space. Unless class list is greater than three, in this case just define taxa to kingdom.

**Studied taxa - order:** If applicable, the order of the taxa studied from iNaturalist taxonomy. If multiple, list all orders, separated by a comma and space. Unless order list is greater than three, in this case just define taxa to class.

**Studied taxa - family:** If applicable, the family of the taxa studied from iNaturalist taxonomy. If multiple, list all families, separated by a comma and space. Unless family list is greater than three, in this case just define taxa to order.

**Studied taxa - genus:** If applicable, the genus of the taxa studied from iNaturalist taxonomy. If multiple, list all genus, separated by a comma and space. Unless genus list is greater than three, in this case just define taxa to family.

**Studied taxa - species:** If applicable, the scientific name of the species studied in the paper from iNaturalist taxonomy. If multiple, list all species, separated by a comma and space. Unless species list is greater than three, in this case just define taxa to genus.

Species of conservation concern: Yes or no if taxon studied is of conservation concern (i.e. threatened, endangered) and this is mentioned in the manuscript.

**Non-native species:** Yes or no if taxon is studied in their non-native range and this is mentioned in the manuscript.

**Paper topic:** Categorically place papers into specific topic categories (see below). If more than one topic is present in the paper, select all that apply. This is in relation to how they used iNaturalist, not all topics mentioned in the paper.

**Topic Notes (if other):** If you select “other” in the paper topics field, use this column to provide a descriptor or list of descriptors to describe the prominent topics of the paper. For this field, use as few words as possible to describe the topic. For example, you could describe a paper that discusses the ethics of authorship when using iNaturalist data as “ethics, authorship.” If more detailed notes are needed, add them to the “Notes” column.

**Analyses conducted:** Categorically place papers into analyses conducted on iNaturalist data (see below). If more than one analyses was conducted on iNaturalist data, select all that apply.

**Analyses Notes (if other):** If you select “other” in the analyses conducted field, use this column to provide a descriptor or list of descriptors to describe the prominent analyses of the paper. For this field, use as few words as possible to describe the topic. For example, you could describe a paper that uses human surveys as “human survey.” If more detailed notes are needed, add them to the “Notes” column.

**iNaturalist data type:** The type of data used from iNaturalist - locational, imagery, date and time, etc (see below for all options). For example, if authors use iNaturalist to create a range map, then they are using observation data. If they are examining images for species traits, then they are primary using the imagery. Choose all data types that apply.

**Data Types Notes (if other):** If you select “other” for iNaturalist data type use this field to describe the other data type. This could be project information, taxonomic backbone, data annotation, etc. Use one or two words to describe the data type. If a more detailed description is needed, use the notes field.

**iNaturalist data role:** The role that iNaturalist data played in the paper. From the main data source to supporting data to descriptive (see iNaturalist_Data_Role table for all possibilities).

**iNaturalist project:** Yes or no if the article uses iNaturalist data from a project or heavily discuss an iNaturalist project.

**No tags because:** If you cannot determine the article tags, use this column to select the reason. The list of reasons can be found in the No_tags_because table. This includes you not having access to the full text article, the article not fitting the screening criteria (scientific paper that is peer-reviewed, full-text article is electronically available, article is in English or Spanish, and article has an iNaturalist component), confusion about how iNaturalist data was used, or article is in Spanish which you cannot understand (In this case, the article will be sent to Flo). You may leave additional information in the “Notes” column. If you were able to determine tags for an article, leave this column blank.

**Notes:** Any additional notes about the paper. If you were unsure about any aspect of tagging, leave a comment here and Brittany will review.

**Entered by:** The person who tagged the article.

**Paper topics**

| **Topic** | **Definition** |
| --- | --- |
| **Species Distribution/Range** | Article defines a species' distribution/range including range shifts using iNaturalist data. This also includes species checklists of a specific area. |
| **Biodiversity/Population Assessment** | Article measures species biodiversity (species richness, abundance, diversity, etc) or assesses a population trend overtime |
| **Species Discovery** | The paper documents the discovery of a new species or rediscovery of thought-to-be extinct species. This includes iNaturalist observations that document a new species/species rediscovery or iNaturalist observations that support the documentation of a new species/species rediscovery. This does not include documentation of a species in a new region, as this would go under “Species Distribution/Range” |
| **Biology/Behavior** | Article studies animal biology, species traits, or behavior. This includes habitat use (when determined via imagery instead of observations overlapping remote sensing data), behavior, and phenology. This also includes studies on evolution or taxonomy where species traits in different regions are assessed. Further this includes interspecies interactions such as mutualism, commensalism, parasitism, or competition. It also includes plant-pollinator associations. |
| **Climate Change/Environmental Impact** | Article uses iNaturalist data to assess climate change or other environmental impacts such as pollution, urbanization on species health, etc. |
| **Data Quality/Comparison** | Article assesses iNaturalist data quality or compares it to other data types or platforms. This includes studies on false identifications, reporting bias, observer behavior, and changes in iNaturalist use. This also includes comparison between iNaturalist data and professional surveys, other citizen science data, museum collections, etc. |
| **Education/Community Engagement** | Article discusses iNaturalist as an education or community engagement tool. |
| **Other** | There is a prevalent topic in the article that does not fit into one of the predefined categories. If you select other, fill in the “Topic Notes (if other)” column with one to three words to describe the topic. If a longer description of the topic is necessary, use the “Notes” column to elaborate. |

# **Analyses**

| Analysis | Definition |
| --- | --- |
| Descriptive | Article uses iNaturalist data for descriptive purposes or presents basic summary statistics. This includes species lists, percentage of observations in a region, iNaturalist user count, or the use of iNaturalist imagery to demonstrate a trait or behavior (unless image tagging or analysis is conducted, in which case the article would go under “Image Analysis”). An article can be tagged as descriptive with another analysis category if the “descriptive” element is not related to the other analysis category. For example, if summary statistics are reported for a species distribution model, the paper would only fall under the “Species Distribution” category. However, if the authors used iNaturalist data to calculate a species distribution model and determine a species list for a region, then “Species Distribution” and “Descriptive” and should be selected. |
| Species Distribution | Article calculates species distribution using a statistical techniques or simple data mapping. This includes ecological niche models and habitat models. |
| Population Measure | Article uses an analysis to calculate species occupancy, abundance, richness, diversity, evenness, or other related population or biodiversity measures. |
| Image Analysis | Analysis that involves the use of images, even if just summary statistics (i.e. based on iNaturalist images, X% of Everglades black racers are grey in color). This could be used to assess color variation in animals, habitat use, animal behavior, interspecies interactions, etc. This does not include simple trait descriptions where articles reference an image to illustrate the trait (i.e., some scorpions are found in burrows, see iNat obs #10293) – this would go under “descriptive.” |
| Review | A literature review paper or overview paper involving iNaturalist. |
| Other | Main analysis in the paper does not fall into one of these categories, use the "Analysis Notes (if other)" to describe. |
| Could Not Determine | The analyses used by the authors was not clearly defined enough to place the article into one of the above categories. |

# **iNaturalist Data Type**

| Data Type | Definition |
| --- | --- |
| Observation Data | Location data, data/time of observations, and observations notes/annotations |
| Imagery | The article uses images for analysis. For example, to quantify trait presence, habitat selection, etc. This also includes assessing computer vision accuracy. |
| User Information | The article uses iNaturalist user information to some degree. This includes studies that filter by observerid, analyze observer trends overtime, etc. |
| Identification Data | The article uses identification data to some degree. For example, to quantify the number of misidentifications of a specific species or to determine the likelihood of identification by taxa, etc. |
| Other | The article uses another data type acquired from iNaturalist |
| None or Could Not Determine | The article did not use iNaturalist data, rather it described iNaturalist as a tool or concept. Or the article was not clear in the data type they used from iNaturalist, so data type could not be determined. |

# **iNaturalist Data Role**

| Data Role | Description |
| --- | --- |
| Main Data Source | iNaturalist is the main data source used in the paper. iNaturalist data may be supplemented with other supporting data types (i.e., covariates), but otherwise it is the sole data of interest to answer the paper's hypotheses |
| Major Data Source | iNaturalist data is required or partially-required to answer the paper's hypothesis, but it is not the sole data of interest. The paper will either not be able to exist without iNaturalist data or it could exist but the paper would be less robust. |
| Minor Data Source | iNaturalist data could be removed from the paper, and the paper could still exist without much change. This includes papers where iNaturalist data is used to explain a phenomenon examined as part of the research questions, it is an additional data source added to a larger database, or iNaturalist is used to inform a study's methods or describe results. |
| Could Not Determine | The role of iNaturalist data is not clear enough to make a determination. |

# **No tags because**

Reasons that a paper could not be tagged. If the paper was able to be tagged, leave this field blank.

| Reason | Description |
| --- | --- |
| Not Relevant | Article is not relevant for this project: Not a scientific paper that is peer-reviewed, not a full-text article that is electronically available, article is not in English or Spanish, or article does not have an iNaturalist component. |
| No Access | You could not access the full-text article. If this is the case, the article should go to the “screened by” person who originally gained access. Once this occurs, this “could not determine because” designation can be removed. |
| Unclear Use of iNaturalist | The article mentions iNaturalist in the methods, but the use of it is too unclear to tag the article. |
| Article is in Spanish | The article is in Spanish. This article will need to be sent to Flo for determination. In this case, ensure the “language” column is correct. |
| Other | There is another reason the article could not be tagged, leave comment in notes. |
