# Supplementary material for: iNaturalist accelerates biodiversity research
Source: Bioscience. 2025 Jul 28;75(11):953–65. doi: 10.1093/biosci/biaf104 (PMC12650526; doi:10.1093/biosci/biaf104)
Supplement: biaf104_Supplemental_Files [file biaf104_supplemental_files.zip › Supplementary_File_2_Mason_et_al.docx]

**Supplementary File 2**

**
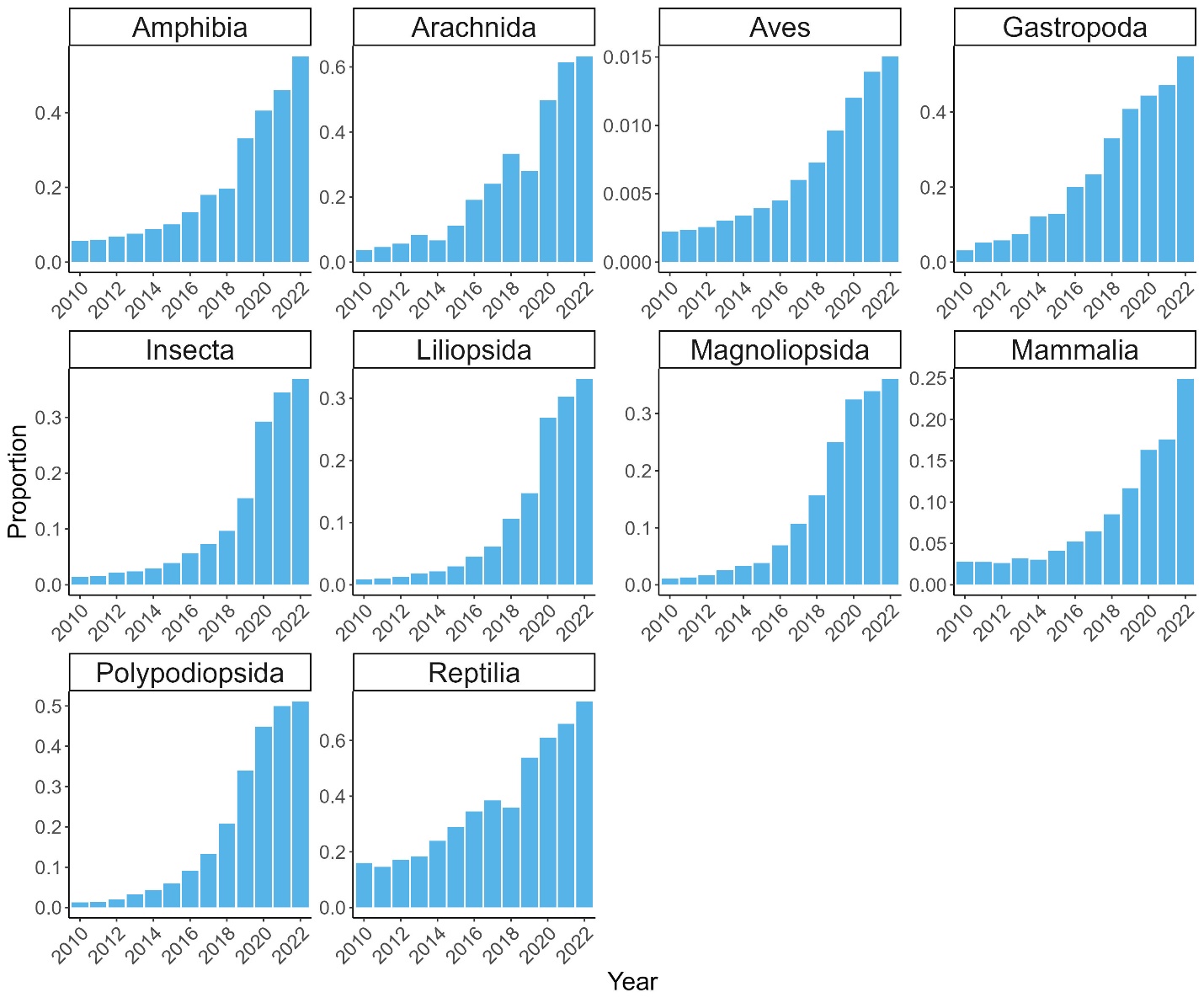
**

**Figure S1.** The proportion of human observation records contributed by iNaturalist to the Global Biodiversity Information Facility over time, grouped by the top ten classes on iNaturalist based on observation count. Because there is variability in the frequency that datasets publish their data, we only analyzed data up to 2022 when the largest GBIF contributing platforms have uploaded their data.

**Alt text:** Ten bar plots, one for each class, showing the growing proportion of iNaturalist in the GBIF database over time.

**
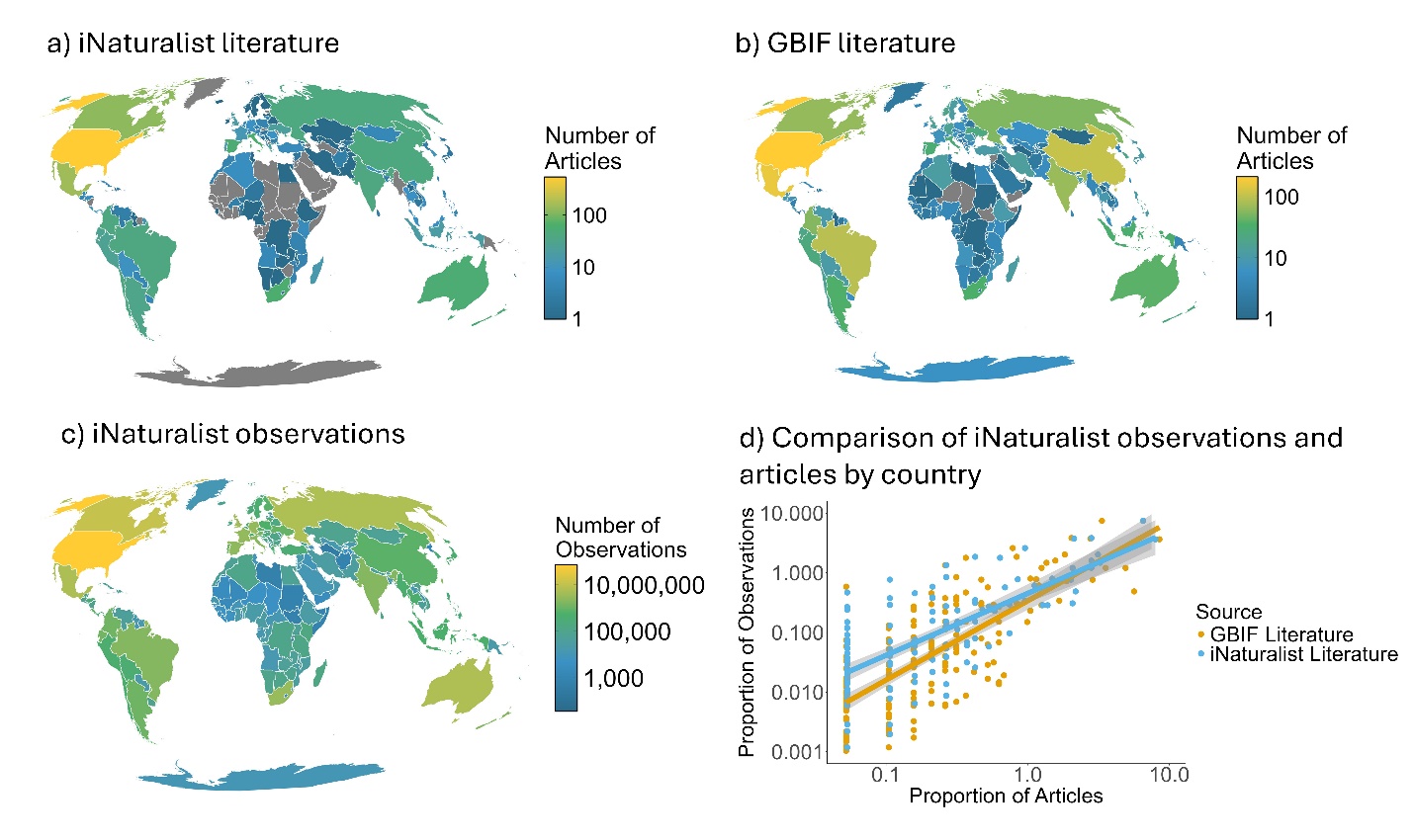
**

**Figure S2.** Geographic distribution of (a) iNaturalist literature, (b) Global Biodiversity Information Facility (GBIF) literature, and (c) iNaturalist observations by country. For the literature, the region represents the article’s study area. Additionally presented is (d) a scatterplot of proportion of articles by proportion of iNaturalist observations for each country. The line represents the linear relationship, and the grey shading is standard error.

**Alt text:** Three global maps showing the proportion of iNaturalist articles, GBIF articles, and iNaturalist observations by country. Additionally displayed is scatterplot with trend lines with grey shading for standard error, that illustrates the positive relationship between the proportion of articles and the proportion of observations, showing that countries with more iNaturalist observations tend to produce more literature using that data.

**
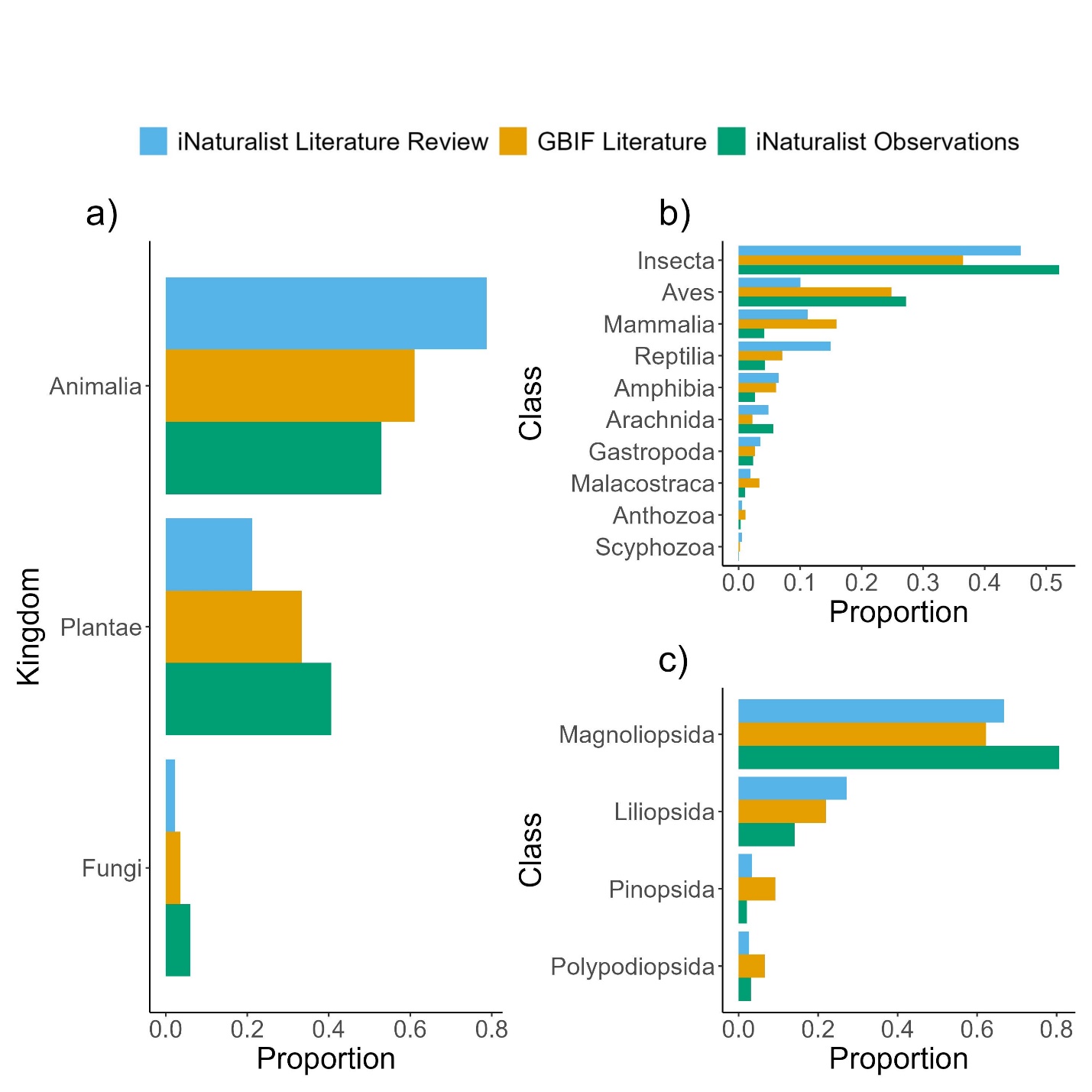
**

**Figure S3.** Taxonomic distribution of articles from iNaturalist literature review, Global Biodiversity Information Facility (GBIF) literature, and iNaturalist observations by (a) kingdom and class for (b) Animalia and (c) Plantae. Displayed are groups that appear in more than 5% of articles in both literature datasets.

**Alt text:** Three bar graphs showing the proportion of taxa from iNaturalist literature, GBIF literature, and iNaturalist observations.


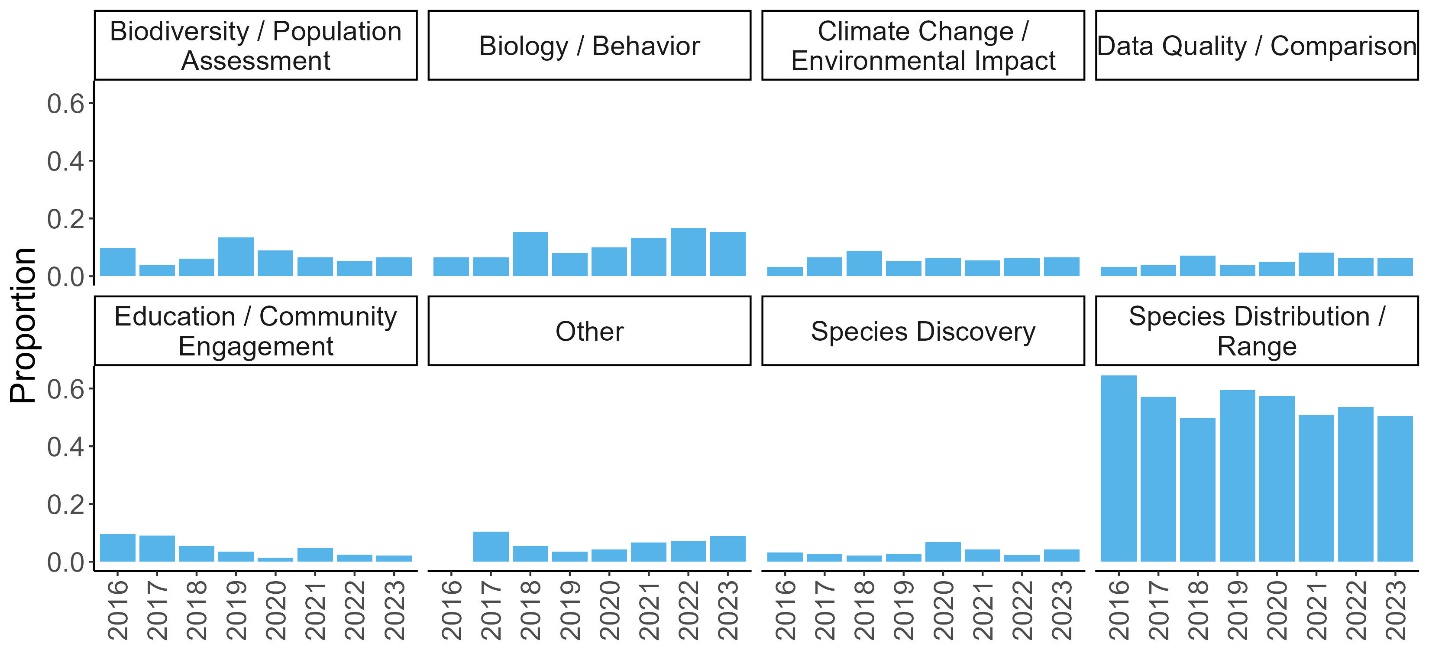


**Figure S4.** Proportion of topics discussed in the literature over time from iNaturalist literature. Data are displayed from 2016 onward, when there were more than 15 articles represented in the iNaturalist literature review.

**Alt text:** A series of bar plots for each topic in the iNaturalist literature and GBIF literature, where individual bar plots show the proportion of each topic over time.


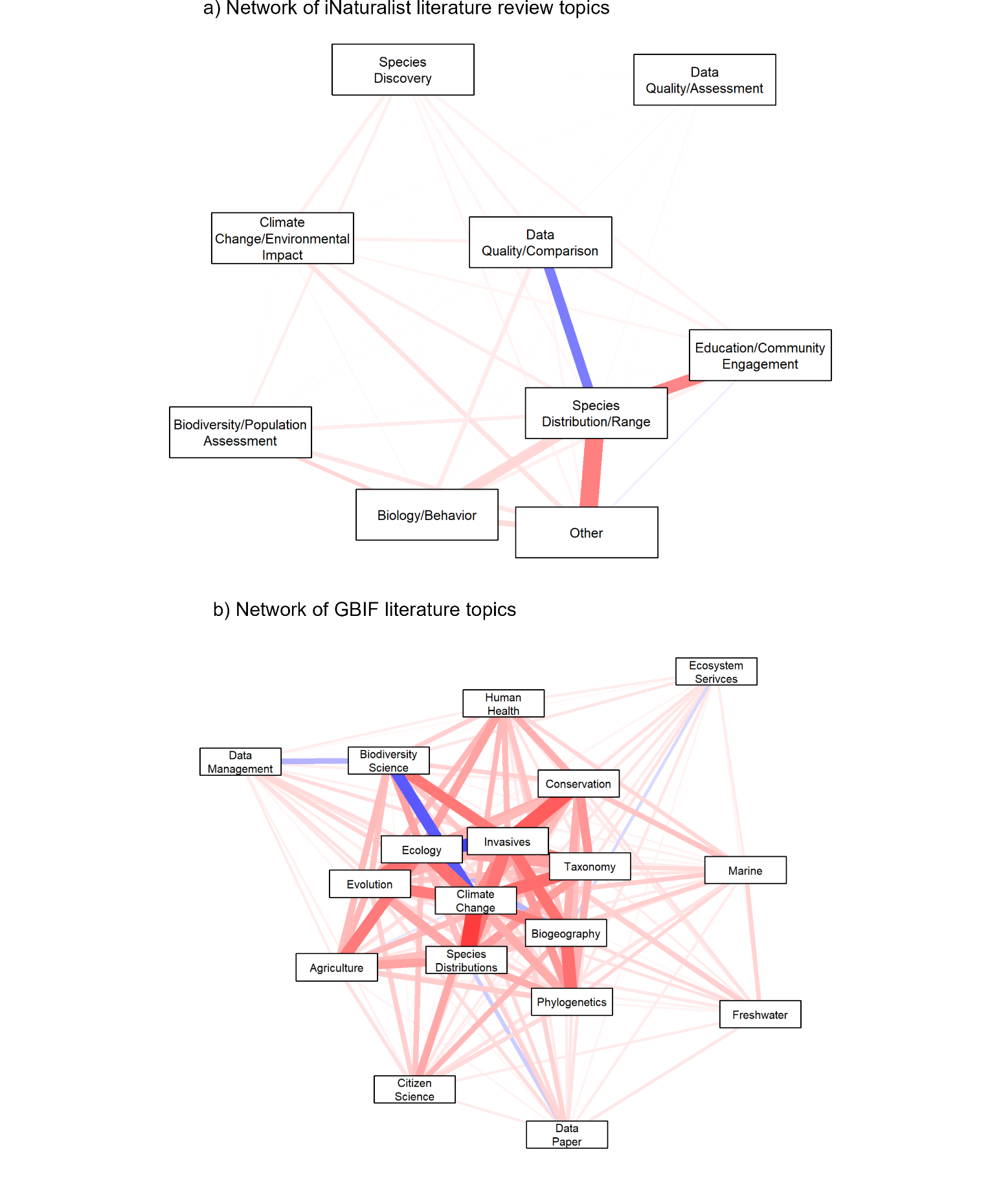


**Figure S5.** Network analysis of topics from (a) iNaturalist literature review and (b) GBIF literature. Boldness of the line and spatial closeness indicates strength of the relationship. Red indicates negative correlation and blue indicates positive correlation. We found mostly negative associations among topics, as 70% of iNaturalist articles and 62% of GBIF literature only fit within a single topic (Figure S1). However, within the GBIF literature, we observed positive associations between *Biodiversity Science* and *Ecology*, *Biodiversity Science* and *Climate Change*, *Ecology* and *Invasives*, and *Data Management* and *Biodiversity Science*. These positive co-occurrences indicate that biodiversity science is often found in conjunction with topics on ecology, climate change, and data management. Additionally, studies on invasive species are often associated with ecology.

**Alt text:** Two network plots, one for iNaturalist literature and one for GBIF literature. In each network, topics are displayed in white boxes, connected to other topics with a red or blue line to depict correlation.


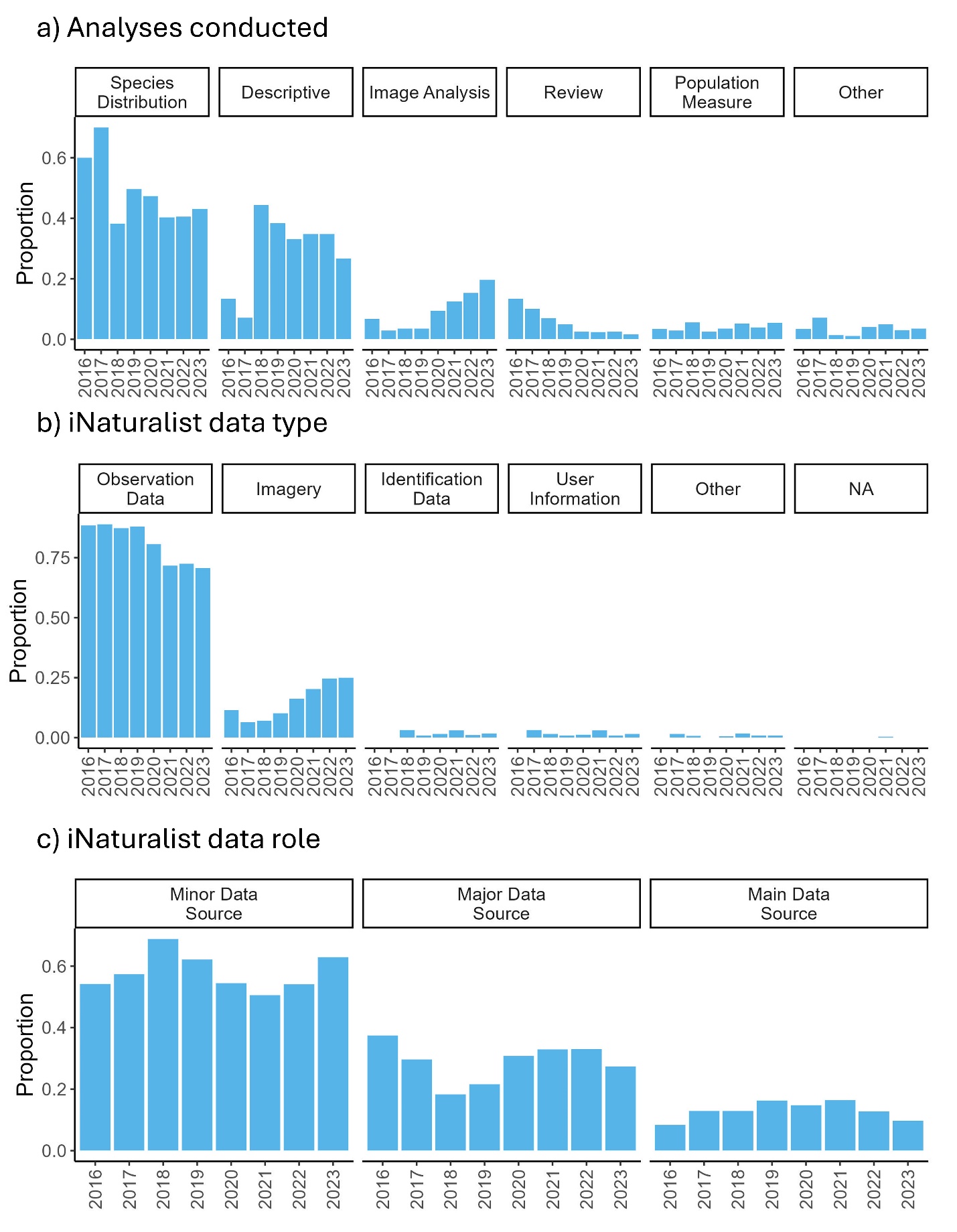


**Figure S6.** Proportion of (a) analyses conducted from iNaturalist data, (b) iNaturalist data type, and (c) iNaturalist data role over time from iNaturalist literature review papers.

**Alt text:** Three sections of bar plots, where each section contains an individual bar plot for (a) analyses conducted (b) iNaturalist data type, and (c) iNaturalist data role. Each bar plot displays the proportion of each item by year from 2016 to 2023. There is an increasing trend in image analyses and image data type, and a decreasing trend in review type papers.

**Table S2.** Count of scientific papers from the iNaturalist literature review and GBIF literature using iNaturalist data compared to the number of iNaturalist observations by country. The residual column represents the residual difference from a log-transformed proportion comparison of the number of articles to observations, based on a linear model (see Figure 3C). Negative residuals indicate a higher proportion of articles published using iNaturalist data than expected given the number of observations, while positive residuals indicate a lower proportion than expected.

| Country | Number of Articles | Number of iNaturalist Observations | Residual |
| --- | --- | --- | --- |
| Afghanistan | 7 | 828 | -4.25 |
| Albania | 8 | 40717 | -1.93 |
| Algeria | 19 | 107464 | -1.82 |
| Angola | 8 | 13702 | -3.01 |
| Antarctica | 9 | 10803 | -3.37 |
| Argentina | 73 | 1135423 | -0.81 |
| Ashmore and Cartier Islands | 51 | 37 | -10.78 |
| Australia | 104 | 6036118 | 0.51 |
| Austria | 7 | 1330807 | 1.70 |
| Bangladesh | 15 | 23023 | -3.12 |
| Belgium | 13 | 432550 | -0.05 |
| Belize | 7 | 93655 | -0.96 |
| Benin | 16 | 72660 | -2.04 |
| Bolivia | 21 | 379244 | -0.66 |
| Brazil | 147 | 1901590 | -0.99 |
| Bulgaria | 6 | 96943 | -0.77 |
| Canada | 220 | 11721764 | 0.42 |
| Chile | 62 | 484236 | -1.50 |
| Colombia | 99 | 1203274 | -1.05 |
| Costa Rica | 27 | 896948 | -0.05 |
| Croatia | 8 | 239931 | -0.15 |
| Denmark | 11 | 1209040 | 1.15 |
| Ecuador | 51 | 1258951 | -0.35 |
| El Salvador | 6 | 63487 | -1.19 |
| Ethiopia | 11 | 26336 | -2.68 |
| Fiji | 6 | 42997 | -1.58 |
| Finland | 9 | 736421 | 0.85 |
| France | 21 | 2807496 | 1.34 |
| Georgia | 8 | 72062 | -1.35 |
| Germany | 24 | 2917601 | 1.25 |
| Greece | 35 | 450349 | -1.00 |
| Guatemala | 12 | 98700 | -1.45 |
| Honduras | 14 | 238851 | -0.72 |
| India | 116 | 1899615 | -0.76 |
| Indonesia | 57 | 536316 | -1.31 |
| Iran | 13 | 30043 | -2.71 |
| Ireland | 7 | 143653 | -0.53 |
| Italy | 90 | 2318108 | -0.30 |
| Japan | 13 | 456326 | 0.01 |
| Kazakhstan | 7 | 84347 | -1.06 |
| Kenya | 6 | 176758 | -0.17 |
| Latvia | 7 | 37188 | -1.88 |
| Lithuania | 6 | 348572 | 0.51 |
| Madagascar | 28 | 152671 | -1.86 |
| Malaysia | 16 | 548744 | -0.02 |
| Mexico | 330 | 5751494 | -0.69 |
| Mozambique | 11 | 34284 | -2.42 |
| Nepal | 19 | 49430 | -2.60 |
| Netherlands | 7 | 610334 | 0.92 |
| New Zealand | 81 | 1903440 | -0.40 |
| Nigeria | 6 | 21795 | -2.26 |
| Norway | 26 | 235224 | -1.35 |
| Panama | 10 | 429507 | 0.21 |
| Papua New Guinea | 6 | 21358 | -2.28 |
| Paraguay | 12 | 21364 | -2.98 |
| People's Republic of China | 152 | 767286 | -1.93 |
| Peru | 51 | 450044 | -1.37 |
| Philippines | 15 | 238352 | -0.79 |
| Poland | 6 | 593775 | 1.04 |
| Portugal | 41 | 1224682 | -0.16 |
| Romania | 19 | 125186 | -1.67 |
| Russia | 99 | 5887187 | 0.53 |
| Singapore | 22 | 462666 | -0.51 |
| South Africa | 103 | 3183765 | -0.12 |
| South Korea | 33 | 363943 | -1.15 |
| Spain | 86 | 2516126 | -0.18 |
| Sweden | 16 | 420635 | -0.28 |
| Switzerland | 6 | 590562 | 1.04 |
| Thailand | 19 | 493335 | -0.30 |
| Trinidad and Tobago | 10 | 91157 | -1.34 |
| Turkey | 17 | 369205 | -0.47 |
| Uganda | 7 | 58476 | -1.43 |
| Ukraine | 49 | 966194 | -0.57 |
| United Kingdom | 35 | 4093712 | 1.21 |
| United States of America | 822 | 71335243 | 0.91 |
| Uruguay | 7 | 85301 | -1.05 |
| Venezuela | 12 | 45223 | -2.23 |
| Vietnam | 7 | 95265 | -0.94 |
